# Aphasia rehabilitation: a narrative review of adjuvant techniques

**DOI:** 10.3389/fnhum.2025.1554147

**Published:** 2025-07-30

**Authors:** Stacy M. Harnish, Courtney C. Jewell, Natalie G. Freitag, Grace E. Terry, Gillian I. Anderson

**Affiliations:** Department of Speech and Hearing Science, College of Arts and Sciences, The Ohio State University, Columbus, OH, United States

**Keywords:** aphasia, adjuvant, treatment, non-invasive brain stimulation (NIBS), aerobic exercise, pharmacotherapy

## Abstract

Adjuvant techniques, or strategies that may be employed alongside language therapy for individuals with aphasia, are increasingly gaining attention for their ability to promote an enhanced brain environment for neuroplasticity. This narrative review describes active ingredients, mechanisms of action, potential modulating factors and evidence for efficacy of non-invasive brain stimulation techniques, such as transcranial direct current stimulation (tDCS) and transcranial magnetic stimulation (TMS); aerobic exercise; intention treatment; and pharmacotherapies, including monoaminergic, cholinergic, glutaminergic, and nootropic medications that have been used in concert with language therapy for aphasia.

## Introduction

Chronic language impairments after brain injury, or aphasia, affect approximately a third of the 25.7 million survivors of stroke worldwide (Ali et al., 2021). Therapy for aphasia either targets restoration of function using principles of neuroplasticity or compensation of function using strategies or devices to assist with communicative effectiveness. Restorative treatments for aphasia have leveraged adjuvant techniques that enhance neuroplasticity through various mechanisms. These techniques may help to create a more favorable environment for the brain to adapt and respond to therapy following injury. Adjuvant techniques for aphasia are particularly important, as widely used treatments have a substantial percentage of non-responders (Efstratiadou et al., 2018; Johnson et al., 2019; Pierce et al., 2019), and thus, people with aphasia could benefit from techniques that enhance traditional therapy.

Language processing in the brain involves several key regions, which are often lateralized to the left hemisphere. According to the Wernicke-Lichtheim model (Lichteim, 1885), the left inferior frontal gyrus (i.e., Broca's area—essential for language production) is connected to the posterior portion of the left superior temporal gyrus (i.e., Wernicke's area—essential for language comprehension) by the arcuate fasciculus. Damage to any of the aforementioned substrates may result in impaired communication. For example, an injury to Broca's area often results in impaired speech production (i.e., Broca's aphasia) whereas damage to the arcuate fasciculus often results in impaired repetition, despite intact comprehension (i.e., conduction aphasia; Bernal and Ardila, 2009). The Wernicke-Lichtheim model provides a foundational, simplified understanding of language processing. However, language processing is a highly interconnected function involving a wider-spread network of brain regions and pathways. Additional substrates and networks of the brain critical for language include, but are not limited to, the angular gyrus, supramarginal gyrus, primary auditory cortex, the default mode network, and various subcortical structures, such as the basal ganglia and thalamus (Chen et al., 2021; Fridriksson et al., 2018c; Gordon et al., 2020). Together, the comprehension and production of language are a complex process that integrates various regions and pathways in the brain. Targeting specific brain regions, pathways, and networks alongside language deficits in speech-language therapy is of utmost importance given the connection, though non-linear, between clinical presentation and the site of neurological injury.

Herein, we review adjuvant techniques that have been reported in the aphasia treatment literature, including non-invasive brain stimulation such as transcranial direct current stimulation (tDCS) and repetitive transcranial magnetic stimulation (rTMS); aerobic exercise such as walking or cycling; intention treatment; and pharmacotherapy in the acute and chronic stages of recovery, including monoaminergic, cholinergic, glutaminergic, and nootrophic medications. Each section will cover the active ingredients of each approach, mechanisms of action, potential modulating factors, and evidence of efficacy.^1^

### Non-invasive brain stimulation

Broadly, the goal of non-invasive brain stimulation (NIBS) in post-stroke language recovery is to enhance behavioral speech-language therapy (SLT) by promoting neuroplasticity. The two most commonly used NIBS techniques are transcranial direct current stimulation (tDCS) and repetitive transcranial magnetic stimulation (rTMS).

tDCS uses saline-soaked sponge electrodes to deliver a constant current of 1–2 mA to the region of interest (Breining and Sebastian, 2020). The polarity is binary such that there is an anodal and cathodal electrode. It is largely agreed that the anodal electrode excites neural activity in the targeted brain region, whereas the cathodal electrode inhibits activity (Crosson et al., 2015). Researchers typically administer anodal tDCS during SLT to the lesioned area (i.e., the left hemisphere), or cathodal tDCS to the contralesional area (i.e., the right hemisphere; Breining and Sebastian, 2020).

rTMS uses a head coil to deliver a changing magnetic field at a high frequency (> 1, 5, or 10 Hz) to excite the targeted region, or at a low frequency (≤1 Hz) to inhibit the region (Crosson et al., 2015; Yoon T. H. et al., 2015). Thus, rTMS differs from tDCS in that rTMS can elicit motor activity, but tDCS cannot. That is, rTMS can directly cause neurons to fire action potentials, whereas tDCS enhances excitability but does not directly elicit motor activity. Both rTMS and tDCS can be administered with or without simultaneous SLT. Although tDCS and rTMS differ in their applications to aphasia therapy, both achieve the same goal of modulating neuronal activity.

#### Active ingredients

NIBS consists of two active ingredients. The first is the ***application of an excitatory or*** ***inhibitory signal to the brain***. In studies employing tDCS, anodal (i.e., excitatory) tDCS is most often used and applied to the site of lesion (Elsner et al., 2020). Although less common, high frequency rTMS can also be used to excite neuronal activity (Zhang et al., 2017). In right-handed individuals, the site of lesion may be the left inferior frontal gyrus, or the left temporoparietal region, broadly (Elsner et al., 2020; Fridriksson et al., 2018b). Through this technique, surviving neurons in the perilesional tissue are stimulated. Conversely, cathodal (i.e., inhibitory) tDCS or low frequency rTMS may be applied to the contralateral, homologous site of the lesion (e.g., right inferior frontal gyrus, right temporoparietal region; Breining and Sebastian, 2020; da Silva et al., 2018; Spigarelli et al., 2024). The rationale behind this method is that inhibition of the right hemisphere will force activation to shift back to the left hemisphere (Ren et al., 2019).

The second active ingredient is ***the use of NIBS with or without concurrent*** ***speech-language therapy (SLT)***. The theory underlying excitatory NIBS usage during SLT is that neurotransmission is facilitated through increasing the neuron's resting potential. Proponents of this theory argue that NIBS is task-dependent, meaning the neurons must be attempting to fire for NIBS to have an augmentative effect (Bikson et al., 2018; Kronberg et al., 2017). As such, tDCS is almost always administered during SLT. However, rTMS is sometimes used in the absence of SLT (Spigarelli et al., 2024).

#### Mechanisms of action

The goal of both tDCS and rTMS is to induce long-lasting changes in cortical excitability to promote language recovery in individuals with post-stroke aphasia. In other words, by increasing long-term potentiation (LTP) or decreasing long-term depression (LTD) in the perilesional tissue, NIBS facilitates more efficient neurotransmission (Kronberg et al., 2017). There are a few leading theories as to how this effect is achieved.

Myelination, a proposed mechanism of LTP, is the process by which the axons of active neurons become surrounded by oligodendroglia cells (Maas and Angulo, 2021). It is dependent upon the release of glutamate onto the N-methyl-D-aspartate (NMDA) receptors of the oligodendrocytes. This process increases the speed at which the cell fires and communicates with its synaptic connections (Lundgaard et al., 2013). Increasing neuronal activity via NIBS may promote myelination, potentially explaining why individuals who receive NIBS during SLT can name pictures with increased accuracy and reduced latency. However, more sensitive measures (e.g., diffusion tensor imaging) are needed to measure myelination to draw more definitive conclusions (Maas and Angulo, 2021).

Alternatively, NIBS may rely on NMDA receptors to change neuronal resting potential (Stagg and Nitsche, 2011). In the animal literature, Kronberg et al. (2017) found that direct current stimulation did not modulate neuronal firing when NMDA receptors were blocked in hippocampal brain slices, despite the presence of strong neural activity. The authors concluded that NMDA receptors are critical in modulating neuronal activity via NIBS. Furthermore, when these NMDA receptors are unopposed, NIBS can either increase firing in the perilesional tissue or decrease firing in the contralesional tissue by altering resting potential (Radman et al., 2009; Rahman et al., 2013). These changes may lead to more efficient neurotransmission and potential restoration of function.

As a whole, understanding of the mechanisms underlying NIBS is still developing. While there is consensus that NIBS influences cortical excitability, the precise mechanism in which this change occurs appears multifaceted and nuanced depending on the technique. For instance, TMS is more likely to indirectly activate neurons at the synapse rather than the axon hillock and maintain precision of stimulation site than transcranial electric stimulation (e.g., tDCS), which is more direct and can result in a shifted stimulation site (Kobayashi and Pascual-Leone, 2003). More research examining the physiological basis of NIBS is needed to guide its clinical usage in people with aphasia (PWA).

#### Potential modulating factors

Some factors that influence the response to NIBS include but are not limited to: lesion size and location, stroke stage, aphasia type and severity, protein production, genetics, and the timing of NIBS usage. To begin, larger lesion sizes have been correlated with a greater degree of improvement following tDCS treatment, which may be secondary to the fact that people with large lesions often present with more severe aphasia (Meier et al., 2019; Norise et al., 2017). Lesion size also influences the site of stimulation (Fridriksson et al., 2018a; Hara et al., 2017). Personalizing stimulation sites based on lesion size and location is a promising approach to expedite treatment outcomes (Fridriksson et al., 2018c).

The time since stroke, or stroke stage, may also modulate the effect of NIBS. In the months following stroke, significant changes occur throughout the brain, such as the lateralization of language functions to the right hemisphere during the acute stages of aphasia recovery (Kiran, 2012; Saur et al., 2006). Whereas, this shift is compensatory, it can become maladaptive when it persists into the subacute and chronic stages (Ren et al., 2019; Turkeltaub, 2015; Yao et al., 2020). Consequently, NIBS is most often employed during the subacute or chronic stage to minimize right hemisphere activation and amplify left hemisphere activation (Breining and Sebastian, 2020). It is generally agreed that most language improvement happens naturally within the first 3 months following stroke (Berthier, 2005; Robey, 1998; Stockert et al., 2020). Thus, NIBS usage may be most appropriate later in recovery as a remediating agent.

Aphasia type and severity are also essential to consider. The right hemisphere is more likely to be recruited in those with more severe aphasia, thus, exciting the right hemisphere may be more appropriate for those with severe language impairments than those with mild or moderate impairments (Liu et al., 2024). Norise et al. (2017) found that those with more severe, expressive deficits demonstrated the greatest improvement following a tDCS + SLT treatment protocol. These findings suggest that NIBS may be more effective for those with more severe deficits, possibly because their recovery progresses slower than expected, requiring additional treatment.

It has been proposed that the release of brain-derived neurotrophic factor (BDNF), and therefore the gene that controls BDNF secretion, modulate the effect of NIBS. BDNF is a protein that is released at the synapse during neural activity (Coelho et al., 2013; Lu, 2003). It is critical for neuroplasticity, and thus language recovery, as it modulates LTP and LTD (Dresang et al., 2022). Those with the typical (val/val) BDNF genotype tend to show greater neuroplasticity, including increased response to rehabilitation and increased ipsilesional cortical activation, in comparison to those with the atypical (Met allele carrier) genotype (Kim et al., 2016). This supports the notion that those with the atypical BDNF genotype have a downregulation in BDNF secretion (Dresang et al., 2022). Furthermore, Fritsch et al. (2010) demonstrated that direct current stimulation was dependent upon BDNF release. This explains the correlation between the typical BDNF genotype, and presumably higher levels of BDNF, and neuroplasticity in tDCS (Fridriksson et al., 2018a) and rTMS studies (Dresang et al., 2022; Uhm et al., 2015). In other words, NIBS is more likely to improve language performance through cortical plasticity in post-stroke individuals who carry the typical BDNF genotype because they express higher levels of BDNF.

Lastly, the time at which NIBS is used can influence its effect on the brain. In clinical practice, NIBS tends to be used *during* SLT, as NIBS is activity-dependent (Fritsch et al., 2010) and task-specific (Bikson et al., 2018; Kronberg et al., 2017). However, the use of NIBS *after* SLT can disrupt therapeutic gains (Rosenkranz et al., 2000). It is suspected that anodal tDCS may strengthen unintended connections, whereas cathodal tDCS may diminish the training-induced activation if administered after training. Taken together, the implementation of precise NIBS timing in clinical settings is crucial.

#### Evidence of efficacy

Preliminary evidence has demonstrated that NIBS, specifically tDCS, may support language recovery in PWA. However, there is significant variability in the stimulation parameters used across studies (i.e., variability in the strength of signal, site of stimulation, etc.). As such, this section will focus on the most common protocols used in NIBS research and their efficacy.

There is good evidence to support the use of anodal tDCS on the left cortex when paired with concurrent SLT. The most recent randomized controlled trial (RCT) investigating anodal tDCS to the left inferior frontal gyrus was conducted by Zheng et al. (2024). They performed a double-blind experiment in which 1 mA was delivered during 20 min of SLT to individuals with chronic aphasia across 5 consecutive timepoints. Compared to sham stimulation, participants who received active tDCS demonstrated greater improvement in name and word-finding. These results corroborate previous studies utilizing left cortex stimulation (Elsner et al., 2020; Fridriksson et al., 2018b).

Fewer studies have investigated the duration of the effect of tDCS. For example, Zheng et al. (2024) measured language abilities through the Western Aphasia Battery- Revised (Kertesz, 2006) immediately after treatment, but did not probe for maintenance. Furthermore, Norise et al. (2017) found no impact of tDCS on measures of fluency at 2-months post-treatment. Thus, although anodal tDCS appears to improve word-finding abilities in the short-term, more research is needed to determine the long-term effect of anodal tDCS on naming and other language skills (Table 1).

**Table 1:** Aphasia sub-types and findings for included tDCS studies.

**Author (year)**	**Study type**	**Sample size**	**Aphasia Sub-type and/or severity level^a^**	**Etiology**	**Findings**
Elsner et al. (2020)	Systematic review and network meta-analysis	*n* = 471 (25 studies)	NR	Unspecified CVA	There was no significant effect of tDCS on functional communication or verb-naming. There was a significant effect of anodal tDCS on noun-naming.
Fridriksson et al. (2018b)	Randomized controlled trial	*n =* 74	Global: *n =* 1 Broca's: *n =* 18 Transcortical motor: *n =* 1 Wernicke's: *n =* 3 Conduction: *n =* 6 Anomic: *n =* 5 Moderate (on average)	Chronic ischemic CVA: *n =* 72 Chronic hemorrhagic CVA: *n =* 2	Anodal tDCS over individualized areas within the left temporal lobe significantly improved picture-naming compared to sham at 1-, 4-, and 24-weeks post-treatment. Participants with milder aphasia had greater picture-naming gains than those with more severe aphasia.
Norise et al. (2017)	Randomized controlled crossover trial	*n =* 9	Non-fluent: *n =* 9 Mild-severe	Chronic ischemic CVA: *n =* NR Chronic hemorrhagic CVA: *n =* NR	tDCS polarity (i.e., anodal or cathodal) was personalized. Participants with more severe aphasia had greater gains in word-level fluency post-treatment and 2-weeks post-treatment.
Zheng et al. (2024)	Randomized controlled trial	*n =* 45	Fluent: *n =* 24 Non-fluent: *n =* 21 Mild-severe	Chronic ischemic CVA: *n =* 34 Chronic hemorrhagic CVA: *n =* 9 Both: *n =* 2	There was a significant effect of anodal tDCS over the left inferior frontal gyrus on aphasia severity, auditory comprehension, and spontaneous speech.

a If the study reported severity levels, these levels are also included. Severity was primarily based on the Aphasia Quotient on the Western Aphasia Battery- Revised (AQ WAB-R; Kertesz (2006). However, Norise et al. (2017) utilized an earlier version of the Kertesz (1982) and the Cookie Theft narrative (Goodglass et al., 2001). Chronic is defined as > 6 months post-CVA. CVA, Cerebrovascular Accident; NR, not reported.

Furthermore, there is mixed evidence supporting the use of low frequency rTMS on the right cortex with or without concurrent SLT. Some studies have demonstrated that this protocol for rTMS can improve language outcomes for people with aphasia (PWA) in the chronic (Hara et al., 2015; Harvey et al., 2017; Rossetti et al., 2019), subacute (Ren et al., 2019; Yoon T. H. et al., 2015), and even acute (Yao et al., 2020) stage of stroke.

First looking at rTMS without SLT, dos Santos et al. (2017) found no difference in picture-naming abilities between TMS and SLT in their RCT involving individuals in the chronic stage of recovery. Furthermore, a meta-analysis by Spigarelli et al. (2024) found that rTMS was only superior to SLT alone for verb-naming at 6–8 months post-treatment in people with chronic stroke.

In studies that have combined rTMS with SLT, the results are also mixed, but more promising. Whereas, Spigarelli et al. (2024) found no significant difference between active and sham rTMS when combined with SLT in their systematic review, a meta-analysis by Yao et al. (2020) found that rTMS + SLT enhanced naming abilities for individuals with acute and chronic stroke more than sham + SLT or SLT alone.

Altogether, rTMS over the right cortex may benefit individuals with acute or subacute stroke when combined with SLT. However, studies should clearly state how they defined stroke stage (i.e., the range of time after stroke) to more clearly investigate this notion. Given the mixed evidence for rTMS in people in the chronic stage, anodal tDCS over the left cortex is favored over low frequency rTMS for this population. More research is needed to determine whether concurrent SLT is a necessary ingredient in rTMS studies, and if rTMS is an effective therapy for language recovery in either condition (Table 2).

**Table 2:** Aphasia sub-types and findings for included TMS studies.

**Author (year)**	**Study type**	**Sample size**	**Aphasia Sub-type and/or severity level^a^**	**Etiology**	**Findings**
dos Santos et al. (2017)	Randomized controlled crossover trial	*n =* 13	Broca's: *n =* 6 Anomic: *n =* 7	Chronic ischemic CVA: *n =* 13	There was no significant difference in picture-naming when anodal tDCS, right hemisphere TMS, and sham were compared.
Hara et al. (2015)	Clinical trial	*n =* 58	Fluent: *n =* 23 Non-fluent: *n =* 27 Mild-severe	Chronic ischemic CVA: *n =* 29 Chronic hemorrhagic CVA: *n =* 20 Chronic subarachnoid hemorrhage: *n =* 1	Right hemisphere rTMS combined with intensive SLT was associated with significantly improved speaking, writing and reading scores, whereas left hemisphere rTMS was associated with significantly improved speaking, writing and listening scores.
Harvey et al. (2017)	Clinical trial	*n =* 9	Non-fluent: *n =* 9 Mild-moderate	Chronic unspecified CVA: *n =* 9	There was a significant effect of right hemisphere rTMS on picture-naming post-treatment, as well as 2- and 6-months post-treatment. Improved naming was significantly associated with recruitment of the right hemisphere.
Ren et al. (2019)	Randomized controlled trial	*n =* 54	Global: *n =* 54 Severe	^c^Subacute (4–12 weeks) unspecified CVA: *n =* 54	There was a significant effect of right hemisphere rTMS combined with SLT on aphasia severity and repetition. Inhibition of the posterior inferior frontal gyrus uniquely resulted in improved spontaneous speech, whereas inhibition of the posterior superior temporal gyrus resulted in improved auditory comprehension compared to sham.
Rossetti et al. (2019)	Case report	*n =* 1	Anomic: *n =* 1	Chronic ischemic CVA: *n =* 1	rTMS over the right inferior frontal gyrus was significantly associated with improved phonemic fluency.
Spigarelli et al. (2024)^b^	Systematic review and meta-analysis	*n =* 68 (10 studies)	Broca's: *n =* 16 Transcortical motor: *n =* 11 Global: *n =* 4 Non-fluent: *n =* 25 Mild-severe	Chronic ischemic CVA: *n =* 7 Chronic hemorrhagic CVA: *n =* 2 Chronic unspecified CVA: *n =* 59	The meta-analysis demonstrated no significant improvement in verb-naming 1-week to 3-months following rTMS over the right inferior frontal gyrus. Significant improvement was only observed at 6–8 months. The systematic review revealed no significant effect of rTMS combined with SLT on verb-naming when compared to sham. Low methodological quality and potential reliability issues of studies included in the meta-analysis are noted.
Yoon T. H. et al. (2015)	Clinical trial	*n =* 20	Non-fluent: *n =* 20	Subacute and chronic; Ischemic CVA: *n =* 11 Hemorrhagic CVA: *n =* 9	Participants who received right hemisphere rTMS combined with SLT significantly improved in repetition and naming. Participants who received SLT-only did not demonstrate significant improvements in any measured language domains.
Yao et al. (2020)^b^	Systematic review & meta-analysis	*n =* 536 (18 studies)	Non-fluent: *n =* 263 (9 studies) Mixed: *n =* 273 (9 studies)	Chronic: *n =* 171 (5 studies) ^c^Acute: *n =* 234 (9 studies) NR: *n =* 131 (4 studies)	Right hemisphere rTMS combined with SLT significantly improved naming, repetition, comprehension, writing and functional communication compared to sham + SLT and SLT-only. rTMS + SLT was favored for both ^c^acute and chronic aphasia.

a If the study reported severity levels, these levels are also included. To determine severity, Hara et al. (2015) utilized the Standard Language Test of Aphasia (Hasegawa et al., 1984); Harvey et al. (2017) used the Boston Diagnostic Aphasia Examination (Goodglass et al., 2001); Ren et al. (2019) and Yoon T. H. et al. (2015) used translated versions of the WAB.

b Data including aphasia type, stroke type (i.e., ischemic or hemorrhagic), stroke stage (i.e., acute, subacute, or chronic) were not universally reported in the articles reviewed by Spigarelli et al. (2024) and Yao et al. (2020). Additionally, only the demographics of participants in the active rTMS condition were reported in Spigarelli et al. (2024) and are included in this table.

c Stroke stage criteria varied by study. Yao et al. (2020) defined < 6 months post-stroke as acute, whereas Ren et al. (2019) defined 4-12 weeks as subacute. CVA, Cerebrovascular Accident; SLT, speech-language therapy; NR, not reported.

Research on NIBS has aimed to determine optimal stimulation parameters, including stimulation site, signal polarity and pattern. For rTMS, there is evidence for high frequency stimulation on the left (Zhang et al., 2017) and right hemispheres (Hara et al., 2017). Additionally, the use of theta burst stimulation, a specific rTMS pulse pattern, has also gained popularity (Breining and Sebastian, 2020), and case studies have offered supporting evidence for its effect on language recovery (Georgiou et al., 2019; Griffis et al., 2016; Vuksanović et al., 2015). For tDCS, the right cerebellum is being considered as a site of stimulation. In two separate studies using anodal and cathodal tDCS, an improvement in language, albeit via different domains, was observed when compared to sham stimulation (Marangolo et al., 2018; Sebastian et al., 2017). Going forward, research on NIBS should directly compare stimulation parameters while also considering that the “optimal configuration” may vary from person-to-person.

### Aerobic exercise

Aerobic exercise is a repetitive physical activity that utilizes the body's metabolic system to generate energy (Millstein et al., 2020). Regular participation in aerobic exercise (e.g., walking, running, cycling) improves the capacity of the cardiovascular system to transport oxygen, providing health benefits (Almeida and Araújo, 2003; Kemi and Wisløff, 2010). Aerobic exercise helps prevent chronic disease, supports brain health, lowers cholesterol, and reduces blood pressure (Lautenschlager et al., 2012; Piercy et al., 2018; Warburton et al., 2006).

Moreover, it can have a positive impact on cognitive functioning (Mayer et al., 2021; Mikkelsen et al., 2017; Smith and Merwin, 2021; Yao et al., 2021), such as executive functioning skills (Guiney and Machado, 2013; Rand et al., 2010), speed of information processing (Quaney et al., 2009), memory (Gómez-Pinilla et al., 2007; Vaynman et al., 2004; Yamada et al., 2002), and language (Cumming et al., 2012; El-Tamawy et al., 2014; Gary and Brunn, 2014; Gomez-Pinilla and Hillman, 2013; Mayer et al., 2021)

#### Active ingredients

***The intensity and duration (i.e., dose) of aerobic exercise*** serve as key active ingredients. Research suggests that programs lasting at least 4 weeks are effective (Moore et al., 2010; Zheng et al., 2016), but longer durations (e.g., 8 weeks or more) are often recommended for optimal improvement (Gezer et al., 2019; Harnish et al., 2018; Potempa et al., 1995). Tracking aerobic exercise dose amongst stroke survivors can include the addition of wearable technology to monitor exercise progress, providing real-time data on physical activity and outcomes (Patel et al., 2012; Powell et al., 2016). However, the intensity and duration of aerobic exercise should be individualized based on each person's functional capacity. That is, the “dosage” of exercise, defined by frequency, intensity, and duration, needs to be tailored to the unique needs and limitations of each individual (Potempa et al., 1996; Winstein et al., 2016). Doing so is essential for maximizing the benefits of aerobic exercise, particularly for PWA. One caveat is that the optimal dosage of exercise required to enhance neuroplastic changes associated with language recovery is unknown at this time.

#### Mechanisms of action

Aerobic exercise is increasingly recognized for its potential to facilitate brain recovery following stroke, particularly in the areas of neuroplasticity and mental health (Medeiros et al., 2020; Penna et al., 2021). It has been linked to the upregulation of BDNF, and thus increased neuroplasticity and management of energy metabolism (Allard et al., 2017; Vaynman et al., 2004; Vaynman and Gomez-Pinilla, 2005). In addition to highly plastic brain environment, increased levels of BDNF are positively associated with enhanced cognitive functions, all of which may support the restoration of language functions in PWA (Cunha et al., 2010; Gómez-Pinilla et al., 2007; Huang and Reichardt, 2001; Vaynman et al., 2004; Yamada et al., 2002).

In a study by Harnish et al. (2018), PWA who participated in a 12-week aerobic exercise program and language therapy demonstrated increased levels of BDNF and improved language performance. However, interpretation of these findings is limited by the fact that data were not collected on participants' genotypes. There are genetic variations in the BDNF gene that control the activity-dependent secretion of BDNF (Egan et al., 2003). Thus, individuals with BDNF polymorphisms (e.g., -met allele carriers) may experience less BDNF upregulation as a result of aerobic exercise, highlighting the importance of patient-specific factors, such as genetics, in response to therapeutic techniques.

PWA are more likely to experience post-stroke depression (PSD) than those without aphasia, which can further challenge rehabilitation and recovery (Zanella et al., 2023). PSD is associated with negative outcomes such as higher mortality rates and decreased quality of life (Medeiros et al., 2020; Robinson and Jorge, 2016). Participation in aerobic exercise positively impacts serotonin and dopamine levels, which are key neurotransmitters involved in mood regulation (Duman et al., 2008), and alleviate symptoms of depression in people with aphasia.

By reducing symptoms of depression, aerobic exercise can indirectly benefit language recovery and overall quality of life. Improved mental health boosts motivation and energy, which can improve engagement in language rehabilitation activities. Together, while the direct impact of aerobic exercise on language recovery remains an area to be investigated, its role in improving mental health, specifically by reducing PSD (Wong et al., 2016), suggests that aerobic exercise may enhance overall recovery.

#### Potential modulating factors

Physical limitations and stroke stage may impact the effectiveness of aerobic exercise. Many stroke survivors face challenges in performing physical exercises due to weakened motor function and fatigue. Appropriate adaptations should come from collaboration with physical and occupational therapists to help meet the unique needs of each individual, enabling them to safely engage in exercise and benefit from its effects (Gaskins et al., 2021; Kwakkel et al., 2004).

Next, the stage of stroke recovery influences the individual's physical capacity, particularly in the early post-stroke period. Peak oxygen consumption is reduced within the first 4 months following a stroke, indicating lower cardiovascular fitness and endurance during this acute stage (Mackay-Lyons et al., 2020; MacKay-Lyons and Hewlett, 2005), likely impacting participation in standard aerobic exercise. As recovery progresses, oxygen consumption typically improves, reflecting the body's gradual adaptation to physical activity. Engagement in a greater frequency and intensity of aerobic exercise may be better suited in more chronic stages post-stroke.

Adaptations to exercise intensity, frequency, and duration must be individualized. Research on stroke rehabilitation shows variability in exercise participation, with frequency ranging from one to five sessions per week (Gezer et al., 2019). Personalized treatment plans that take into account the patient's stage of recovery, physical capabilities, and overall health, will ensure that aerobic exercise remains both effective and safe.

#### Evidence of efficacy

Preliminary evidence suggests that aerobic exercise offers benefits when combined with traditional SLT (Harnish et al., 2018; Medeiros et al., 2020). For example, Yeh et al. (2019) found that aerobic exercise in stroke patients with mild cognitive impairment in combination with computerized cognitive training led to better cognitive and functional outcomes compared to the control group. Moreover, Harnish et al. (2018) found significant positive effects of aerobic exercise on post-stroke language recovery. These more recent findings build upon earlier studies that also found a relationship between aerobic exercise and language recovery following stroke (Cumming et al., 2012; Rand et al., 2010)

Though, the conclusions of these studies and others similar (Globas et al., 2012), are limited by small sample sizes and reduced insight into long-term effects of aerobic exercise on aphasia recovery, as opposed to more global improvement, such as reduced levels of depression.

Overall, there is modest evidence to support aerobic exercise as an adjuvant to aphasia rehabilitation (Cumming et al., 2012; El-Tamawy et al., 2014; Gary and Brunn, 2014; Gomez-Pinilla and Hillman, 2013; Guiney and Machado, 2013; Harnish et al., 2018; Rand et al., 2010). Larger sample sizes, investigations into the mechanism of action, and further exploration of the optimal dosage and timing of exercise are critical future directions (Table 3).

**Table 3:** Aphasia sub-types and findings for included aerobic treatment studies.

**Author (year)**	**Sample size**	**Aphasia sub-type**	**Etiology**	**Findings**
El-Tamawy et al. (2014)	*n =* 30	NR	Ischemic CVA: *n =* 30	The experimental group experienced enhanced cognitive function and increased BDNF levels following participation in aerobic exercise 3 times a week for 8 weeks.
Gezer et al. (2019)	*n =* 50	NR	CVA: *n =* 50	Participants in the experimental group experienced significant improvements in aerobic capacity and mood compared to the conventional group
Globas et al. (2012)	*n =* 38	NR	Cortical CVA: *n =* 19 Subcortical CVA: *n =* 17	Stroke survivors 60 < who participated in a 3-month high-intensity aerobic treadmill program experienced significant improvements in cardiovascular fitness, walking endurance, gait speed, balance, and quality of life.
Harnish et al. (2018)^a^	*n =* 7	Broca's: *n =* 2 Conduction: *n =* 1 Anomic: *n =* 2 Wenicke's: *n =* 1 Transcortical motor: *n =* 1	Ischemic or hemorrhagic CVA: *n =* 7	Group-level comparisons showed that the group that participated in exercise demonstrated a greater overall increase in effect size.
Moore et al. (2010)	*n =* 20	NR	Ischemic CVA: *n =* 10 Hemorrhagic CVA: *n =* 10	The experimental group saw significant increases in daily stepping activity and gait efficiency despite previously plateauing during recovery.
Rand et al. (2010)	*N =* 11	NR	Ischemic CVA: *n =* 9 Hemorrhagic CVA: *n =* 2 Right hemisphere: *n =* 7 Left Hemisphere: *n =* 4	The experimental group experienced improvements in memory and executive function after participating in exercise 2 h and recreation 1 h weekly for 6 months.
Yeh et al. (2019)	*n =* 30	NR	Ischemic or Hemorrhagic CVA: *n =* 30	The experimental group experienced significant improvements in cognitive endurance and mobility, especially when aerobic exercise was combined with computerized cognitive training.

a To determine aphasia severity, Harnish et al. (2018) reported the Aphasia Quotient on the Western Aphasia Battery—Revised [AQ, WAB-R; Kertesz (2006)]. CVA, Cerebrovascular Accident; NR, Not reported.

### Intention treatment

Intention is the selection for execution and initiation of a specific action. Crosson et al. (2005) suggest that non-fluent aphasia is a “disorder of intention,” as individuals with non-fluent aphasia present with difficulty initiating verbal language output. This may suggest that the underlying language deficits may be more related to the selection and initiation of words. Intention treatment aims to re-map language production to undamaged brain areas that support intention movements. This approach leverages neural pathways for intention and re-lateralizes language production to the right frontal lobe through behavioral manipulations.

#### Active Ingredients

Intention treatment relies on two primary active ingredients. The first is initiating a ***complex left-hand movement within the patient's left hemispace*** (i.e., the left side of the visual field; Crosson et al., 2005), motivated by research indicating that individuals with lesions perform better on language tasks when their attention is directed toward the patient's ipsilesional space (Coslett, 1999; Dotson et al., 2008). For instance, individuals with left parietal lesions demonstrated improved language performance when the stimuli were placed in their left hemispace. During intention treatment, participants first lift a lid of a box with their left hand to initiate the presentation of a stimulus item (i.e., *initiating* the treatment). This movement engages ipsilesional pathways and primes intention mechanisms in the right hemisphere. The second active ingredient is ***pairing a verbal output naming task with*** ***the left-hand movements***. If participants responded to the stimulus correctly, they moved forward to the next trial with no left-hand movements. If incorrect, they repeated the correct stimulus following a therapist's cue while making circular hand gestures (Crosson et al., 2007). The coordinated movement (i.e., left-hand movement and output naming task) primes the intention mechanisms in the right hemisphere and facilitate the re-lateralization of language functions (Picard and Strick, 1996).

#### Mechanisms of action

The primary mechanism of action for intention treatment is the re-lateralization, or the shift, of language function from the dominant language hemisphere (i.e., the left) to the nondominant hemisphere (i.e., the right; Crosson, 2008), which is facilitated by the engagement of intentional motor behaviors (i.e., complex left-hand movements). When intentional motor movements are performed, the medial frontal cortex, lateral frontal structures, and basal ganglia are activated (Crosson et al., 2005; Heilman et al., 2003). These substrates play crucial roles in the initiation and execution of motor movements.

The pre-supplementary motor area (pre-SMA) in the medial frontal cortex is involved in motor planning and intention. It is also activated during word generation (Crosson et al., 2001; Picard and Strick, 1996), and damage to this area may help explain a form of non-fluent aphasia (i.e., impaired word generation, intact comprehension/repetition; Beeson and Bayles, 1997). Previous research has suggested a disconnect between the language production processes (e.g., repetition) and the language initiation processes (e.g., word generation; Richards et al., 2002). The complex left-hand movements activate the right pre-SMA, which is near to regions controlling these movements. The proximity suggests that these movements may prime the right hemisphere's intention mechanisms, thereby enhancing word production (Picard and Strick, 1996). The right pre-SMA is intimately linked with the right lateral pre-frontal cortex, an area of the brain responsible for executive functions and cognitive tasks (Matsuzaka et al., 1992; Picard and Strick, 1996). The treatment's effectiveness is based on the assumption that left-hand movements prime the right lateral frontal mechanism, enhancing word generation adults with aphasia (Crosson, 2008).

#### Potential modulating factors

The individual assessment of lesion characteristics, such as size and location, is necessary to determine the potential benefits of intention treatment. Lesion characteristics may influence the availability of perilesional tissue and the integrity of subcortical structures, such as the basal ganglia, which are essential for neuroplasticity (Demarco et al., 2022; Knopman et al., 1984; Parkinson et al., 2009). In patients with aphasia, larger lesions reduce the amount of perilesional tissue for neuroplastic reorganization, limiting the left hemisphere's capacity to recover language ipsilaterally and increasing the reliance on the right hemisphere (Blank et al., 2003). Patients with larger left-hemisphere lesions may benefit more from intention treatment, as this approach could help re-lateralize language functions to brain regions better suited for neuroplastic recovery (Crosson et al., 2007). Conversely, smaller lesions often result in more healthy, perilesional tissue (Demarco et al., 2022; Kiran and Thompson, 2019), allowing local reorganization in the left hemisphere and reducing the need for the compensatory use of the right hemisphere.

The location of the lesion and extent of subcortical damage can influence the efficacy of intention treatment (Kiran and Thompson, 2019; Payabvash et al., 2010; Stockert et al., 2020). Lesions in the frontal and pre-SMA regions of the left hemisphere may benefit more from engaging the contralateral regions (i.e., intention treatment; Picard and Strick, 1996; Sailor et al., 2003). Lesions sparing the basal ganglia and thalamus have been reported to facilitate the natural re-lateralization of language functions even in the absence of explicit behavioral modifications (Crosson et al., 2005). For example, Crosson et al. (2005) found that one participant showed re-lateralization to the right frontal mechanisms after treatment, while the other participant showed re-lateralization before treatment due to intact basal ganglia and thalamus substrates. It is hypothesized that if these substrates are damaged (i.e., thalamus and basal ganglia), the ability to re-lateralize language function may be impeded (Crosson et al., 2005; Kim et al., 2002).

Importantly, Researchers must consider both the size and location as potential modulating factors. For example, a small lesion of the basal ganglia can impede the shift in language function to the right hemisphere, despite the lesion size being small (Crosson, 2008). Thus, while individuals with larger lesions may benefit more from intention treatment, the extent of subcortical damage can also modulate the effects.

#### Evidence of efficacy

Initial studies show that intention treatment is effective for people with chronic, non-fluent aphasia (Crosson et al., 2007; Richards et al., 2002). Moreover, participants re-learned words at a quicker rate during the intention treatment as opposed to the experimental control, particularly for those with moderate to severe word-finding deficits. However, fewer individuals with profound word-naming deficits demonstrated meaningful improvements. Table 4 presents aphasia sub-types and severity levels for included experimental studies exploring the efficacy of intention treatment. Together, results suggest that individuals with moderate-severe anomia demonstrate the greatest benefits from intention treatment.

**Table 4:** Aphasia sub-types and findings for included intention treatment studies.

**Author (year)**	**Sample size**	**Aphasia Sub-type and/or severity level^a^**	**Etiology**	**Findings**
Altmann et al. (2014)	*n =* 7	Broca's: *n =* 2 Conduction: *n =* 4 Anomic: *n =* 1	Ischemic CVA: *n =* 5 Hemorrhagic CVA: *n =* 2	Experimental group produced more words following treatment than the control group. Intentional left hand gestures significantly impacted generalization to discourse
Benjamin et al. (2014)	*n =* 7	Broca's = 2 Conduction = 4 Anomic = 1	Ischemic CVA: *n =* 5 Hemorrhagic CVA: *n =* 2	The experimental group had better generalization than the control group. Right lateralization was due to the intention gesture.
Crosson et al. (2005)	*n =* 2	Nonfluent: *n =* 2 Moderate anomia: n =2	Ischemic CVA: *n =* 2	Both participants showed response to the intention treatment.
Crosson et al. (2007)^b^	*n =* 34	Moderate: *n =* 12 Severe: *n =* 11 Profound: *n =* 1	Ischemic or hemorrhagic CVA: *n =* 34	Participants with moderate to severe word-finding deficits showed treatment gains.
Crosson et al. (2009)	*n =* 5	Broca's: *n =* 2 Anomic: *n =* 2 Mild Broca's: *n =* 1, Moderate: *n =* 3 Profound: *n =* 2	CVA: *n =* 5	Four of the five participants improved after treatment. The participant who did not improve had profound impairments.
Ferguson et al. (2012)^c^	*n =* 4	Transcortical Motor: *n =* 1 Severe Broca's: *n =* 2 Conduction: *n =* 1	CVA: *n =* 3 AVM rupture: *n =* 1	Participants with mild-moderate aphasia improved verbal production with intention gesture treatment.
Richards et al. (2002)	*n =* 3	NR	CVA: *n =* 3	All participants demonstrated significant increase in naming improvements.

a For clinical trials with an experimental and control group, only participant demographics for the experimental group are provided. This is to facilitate the evaluation of results in conjunction with the type of patients that participated in the intervention group. If the study reported severity levels, these levels are also included. To determine severity and/or subtype, Altmann et al. (2014), Benjamin et al. (2014), Crosson et al. (2005), Crosson et al. (2009), and Ferguson et al. (2012) used the Western Aphasia Battery (WAB; Kertesz, 1982).

b Severity was based on performance on a list of 40 words. Authors determined cut-off scores following participation.

c Aphasia subtypes were provided before and after treatment. Aphasia sub-types before intervention are reported. AVM, Arterial-venous malformation; CVA, Cerebrovascular Accident; NR, Not Reported.

Undeniably, the ultimate goal of SLT is not to solely name black-and-white images but to generalize language outcomes from speech therapy to communicate effectively within one's environment (Wright and Shisler, 2005). Mixed results have been presented on the effectiveness of intention techniques in response generalization (i.e., from trained items to untrained items; Crosson et al., 2007, 2009; Ferguson et al., 2012). Notably, Altmann et al. (2014) demonstrated that individuals within an intentional gestures group demonstrated large improvements in discourse tasks, suggesting the presence of stimulus generalization (i.e., from trained tasks to untrained tasks), though this finding has yet to be replicated.

The studies above demonstrate the intention treatment can enhance word production but lack evidence on whether the treatment engages the right lateral frontal mechanisms. As mentioned earlier, Crosson et al. (2005) found that one patient (*n* = 2) demonstrated re-lateralization to the right frontal mechanisms after the treatment. Later studies with larger sample sizes, greater experimental control, and the completion of imaging with control participants supported the hypothesis of re-lateralization to the right lateral frontal mechanism after the intention treatment (Benjamin et al., 2014; Crosson et al., 2009).

Taken together, there is ample evidence to support intention treatment as an adjuvant to aphasia rehabilitation by enhancing word production and supporting re-lateralization to the right frontal brain mechanisms. However, the foundational studies exploring intention treatment papers are relatively dated. Despite its therapeutic efficacy, it serves better as a theoretical foundation by informing clinical rehabilitation research that laterality can play a vital role in language recovery.

### Pharmacotherapy

Medications are another adjuvant strategy studied to enhance the effects of aphasia therapy. If the goal of adjuvants is to create a better neural environment for plasticity to subsequently engage through the experience of aphasia therapy, then medications are a logical choice to study. There have been no medications approved by the US Food and Drug Administration for the treatment of aphasia. Below we outline the active ingredients and mechanisms of action in medications studied during both the acute and chronic phases of aphasia recovery. Since classes of medications have different mechanisms of action, we describe the active ingredients (i.e., medications) and mechanisms of action together for each type of medication.

### Acute phase pharmacotherapy

There is limited research to date on pharmacological adjuvants to aphasia treatment in the *acute* phase of recovery. Table 5 lists medication adjuvants and their mechanisms of action.

**Table 5:** Medication adjuvants to aphasia therapy.

**Medication class**	**Medications**	**Mechanism of action**	**FDA approved to treat**
Monoaminergic selective serotonin reuptake inhibitors (SSRIs)	Fluoxetine (Prozac), Escitalopram (Lexapro), Fluvoxamine (Luvox)	Increases levels of serotonin	Depression, Anxiety, other mood disorders
Monoaminergic amphetamines	Dextroamphetamine (Adderall)	Increases levels of dopamine and norepinephrine	ADHD
Monoaminergic dopamine agonists	Bromocriptine (Parlodel)	Acts on dopaminergic neurons to activate the prefrontal cortex	Parkinson's disease
Monoaminergic Norepinepherine reuptake inhibitors	Atomoxitine (Strattera)	Increases dopamine and norephinerine levels in the frontal lobes	ADHD
Cholinergic Acetylcholinesterase inhibitors (AChEIs)	Donepezil (Aricept), Galantamine (Reminyl, Razadyne)	Prevents breakdown of acetylcholine	Alzheimer's disease
Glutaminergic	Memantine (Namenda)	Blocks NMDA receptor to reduce glutamate excitotoxicity; increases levels of BDNF	Alzheimer's disease
Nootrophic	Piracetam	Modulates AMPA and NMDA receptors, increases availability of acetylcholine	Not FDA approved in the US. Used for Alzheimer's disease in other countries.

NMDA, N-methyl-D-aspartate receptor; BDNF, Brain-derived neurotropic factor; AMPA, α-amino-3-hydroxy-5-methyl-4-isoxazolepropionic acid receptor.

#### Active Ingredients and Mechanisms of Action

##### Thrombolytic medications

As the standard of care for acute ischemic stroke, thrombolytic medications (e.g., Alteplase and Tenecteplase) are a first-line treatment to reduce the amount and extent of a lesion. Mechanical thrombectomy, also a first-line treatment for acute stroke, is outside the scope of this review.

##### Monoaminergic medications

Monoaminergic medications target various neurotransmitter systems involved in mood, cognition, and behavior. By increasing levels of serotonin, SSRIs improve the brain environment for experience-dependent neuroplasticity (Stockbridge, 2022). Selective serotonin reuptake inhibitors (SSRIs), such as fluoxetine (i.e., Prozac), escitalopram (i.e., Lexapro), and fluvoxamine (i.e., Luvox) are commonly prescribed for mood disorders such as depression and anxiety and have been studied for post-stroke aphasia (Hillis et al., 2018); improvements in post-stroke depression could also improve cognitive and language outcomes (Robinson and Jorge, 2016).

##### Nootrophic medications

Piracetam is broadly categorized as a nootropic medication, or a cognitive enhancer. Its mechanism of action related to aphasia treatment is somewhat unclear. Cichon et al. (2021) state that it is a GABA derivative, which has inhibitory properties, but the primary mechanism of action is thought to be related to modulation of AMPA and NMDA receptors that are important for long-term potentiation, and thus, neuroplasticity. Piracetam has also been associated with the cholinergic system by increasing the availability of acetylcholine in certain brain regions (Kishore and Parle, 2009).

### Chronic phase pharmacotherapy

#### Active ingredients and mechanisms of action

##### Monoaminergic medications

Monoaminergic medications, including amphetamines, dopamine agonists, and norepinephrine reuptake inhibitors, work on the neurotransmitter systems associated with serotonin, dopamine, and norepinephrine, and enhance neuroplasticity.

Norepinepherine reuptake inhibitors work by increasing dopamine and norepinephrine levels in the frontal lobe, which improves executive function, attention, and language processing (Park et al., 2022). Atomoxitine (i.e., Strattera) is a norepinephrine reuptake inhibitor that is commonly used for treatment of attention deficit hyperactivity disorder but may improve cognitive functions that underly language (Yamada et al., 2016).

Amphetamines (i.e., stimulants) increase the concentration of neurotransmitters, such as dopamine and norepinephrine, at the synapse. There is some evidence from studies in the animal literature that amphetamines can assist with neural sprouting and synaptogenesis after stroke (Feeney et al., 1982; Goldstein and Davis, 1990; Hurwitz et al., 1991)

Bromocriptine is a dopamine agonist. The mechanism of action for dopamine agonists is stimulation of the dorsolateral frontostriatal circuit which leads to activation of the prefrontal cortex, an area important for executive functions and language processing. Bromocriptine is thought to act on dopaminergic neurons that project to the basal ganglia, supplementary motor area, and anterior cingulum (Stockbridge, 2022).

Atomoxetine is a norepinephrine reuptake inhibitor that has shown potential in improving comprehension in individuals with cognitive impairment post stroke (Yamada et al., 2016). Norepinephrine reuptake inhibitors increase norepinephrine and dopamine levels to activate the prefrontal cortex (Park et al., 2022).

##### Cholinergic medications

Cholinergic medications, or Acetylcholinesterase inhibitors (AChEIs), are medications often used in managing Alzheimer's disease (AD). AChEIs, such as donepezil and galantamine inhibit the breakdown of acetylcholine, a neurotransmitter that is vital for cognitive functions like learning, memory, and attention (Berthier et al., 2006). Language deficits that occur in post-stroke aphasia and other vascular etiologies of cognitive impairment are thought to be impacted by disruptions in the cholinergic system (Dávila et al., 2023). The lateral cholinergic pathway supplies cholinergic innervation to language areas in the brain and is particularly vulnerable to post-stroke lesions. Thus, preventing the breakdown of acetylcholine boosts cholinergic activity which may assist with learning, memory, attention, and language functions (Dávila et al., 2023).

##### Glutaminergic medications

Glutaminergic medications work to restore balance in the glutaminergic system that is often disrupted post-stroke. Memantine, a glutaminergic medication used in the treatment of Alzheimer's disease, works by blocking the N-methyl-D-aspartate (NMDA) receptor, a type of glutamate receptor. Memantine reduces glutamate excitotoxicity, thereby potentially promoting neuroplasticity (Barbancho et al., 2015; Gawande et al., 2024) and increases levels of BDNF, which contributes to neuronal survival, growth, and synaptic plasticity (Dávila and Berthier, 2024).

#### Potential modulating factors

The effectiveness of monoaminergic and cholinergic agents in aphasia is likely influenced by individual patient and lesion characteristics, such as age, overall cognitive abilities, co-occurring medical conditions, and lesion size that potentially impact response to treatment. Some medications may be more appropriate for an individual depending on their type of aphasia and/or severity of aphasia. The pairing of a medication with those patients who would most benefit from the medication's mechanism of action needs further investigation; genetics may play a role in the probability of responsiveness (Di Piño et al., 2016). Finally, as an adjuvant, medications that are combined with language therapy depend in part on the language therapy itself. That is, the effectiveness of the pharmacological agent in assisting with aphasia may be modulated in part by the intensity and/or individualization of language therapy to maximize the potential for neuroplastic changes.

#### Evidence of efficacy

##### Monoaminergic medications

*SSRIs.* Tanaka et al. (2004) found in a double-blind, randomized, crossover study, that 10 patients with fluent aphasia improved on picture naming and exhibited decreased perseverations after taking fluvoxamine for 4 weeks. Corroborating these findings, a cross-sectional study of individuals with chronic aphasia showed that those who took SSRIs for the first 3 months after stroke demonstrated better picture naming ability and provided more detailed information during picture description than those who did not take SSRIs, but had similar aphasia severity, depressive symptoms, and lesion characteristics (Hillis et al., 2018). However, a meta-analysis of three randomized controlled trials including almost 6000 individuals who were post stroke found that fluoxetine use daily for 6 months did not significantly improve functional communication abilities using the Stroke Impact Scale (SIS) compared to a placebo (Mead et al., 2024). It is important to note that the outcome measures in these trials were functional communication and did not include a thorough language assessment. Thus, there is some data to suggest that SSRI use may be promising in the treatment of post-stroke aphasia, but larger clinical trials with less extensive language testing showed that functional language, using broad measures, did not benefit from SSRI use. Therefore, additional studies with larger sample sizes that include thorough assessment of language abilities are warranted. See Table 6 for a summary of the pharmacological studies reviewed.

**Table 6:** Aphasia sub-types and findings for included pharmacological studies.

**Author (year)**	**Medication and dosage**	**Study type**	**Sample size**	**Aphasia sub-type and/or severity level**	**Etiology**	**Findings**
**Monoaminergic medications**						
Tanaka et al. (2004)	SSRI- Fluvoxamine (dosage NR)	Double-blind, randomized, crossover	*N* = 10	“non-severe” fluent aphasia; Wernicke's aphasia or jargon aphasia	Single left CVA	Improved picture naming and decreased perseverations after taking fluvoxamine for 4 weeks
Hillis et al. (2018)	SSRIs	Two arms with SSRI use: one longitudinal and one cross-sectional	*n* = 30 for longitudinal *n* = 43 for cross sectional	Chronic aphasia Longitudinal NR Cross-sectional group mean WAB AQ 74.5 ± 30.9	left hemisphere ischemic stroke	Better picture naming and more detailed information during picture description by people who took SSRIs for the first 3 months after stroke than by those who did not take SSRIs.
Mead et al. (2024)	Fluoxetine 20 mg daily for 6 months	Individual patient data meta-analysis of combined dataset for three RCTs	*N* = 1,250 (637 drug, 613 placebo)	NR	2–15 days post-stroke	No difference in functional communication using Stroke Impact Scale (SIS) compared to placebo.
Yamada et al. (2016)	Atomoxetine 120 mg daily before therapy (40 mg up-titrated to 120 mg.)	Case series	*N* = 4	Motor-dominant aphasia; 2 mild, 1 moderate, 1 severe aphasia	2 participants with left MCA infarcts; 2 participants with left putamen hemorrhages	Improved language ability (Token test, repetition and sequential commands categories of the WAB) and increased blood flow on SPeCT in left IFG.
**Amphetamines**						
Keser et al. (2017)	Dextroamphetamine, 10 mg	Proof of concept, double blind, crossover study with 7–10 days washout; drug + tDCS + SLT for one session; placebo + tDCS + SLT for one session	*N* = 10	Chronic non-fluent aphasia; 9 with Broca's aphasia, 1 with transcortical motor aphasia	Left MCA infarct	Dextroamphetamine + tDCS + SLT elicited significantly greater gains on WAB-R language quotient and aphasia quotient than placebo + tDCS + SLT.
Walker-Batson et al. (2001)	Dextroamphetamine, 10 mg	Prospective, double-blind, randomized, placebo controlled, SLT, 10 sessions	*N* = 21 (12 drug, 9 placebo)	Moderate-severe aphasia	Acute non-hemorrhagic infarct; 16–45 days post-stroke	Dextroamphetamine group showed significantly greater gains than placebo group after therapy. Eighty-three percentage of dextroamphetamine group showed clinically meaningful change as compared to 22% of the placebo group.
**Dopamine agonists**						
Bragoni et al. (2000)	Bromocriptine, 30 mg daily for 18 weeks	Double-blind, placebo-controlled	*N* = 11	Chronic non-fluent aphasia; 2 severe Global; 3 severe Broca's; 3 moderate Broca's; 3 mild Broca's	Post-stroke	High doses of bromocriptine improved reading comprehension and repetition, and verbal latency (time to begin speech production); however 6 of 11 participants dropped out or were withdrawn due to side effects of the bromocriptine, adverse events (seizure, atrial fibrillation, visual hallucinations) or low compliance.
Ashtary et al. (2006)	Bromocriptine, 10 mg daily for 4 months (2.5 mg up-titrated to 10)	Randomized, double-blind, placebo-controlled	*N* = 38 (19 drug, 19 placebo)	nonfluent	Post-stroke; acute	No significant differences between bromocriptine and placebo groups
Seniów et al. (2009)	Levodopa, 100 mg	Prospective, randomized, placebo-controlled, double-blind, before SLT 5 days/week for 3 weeks	*N* = 39 (20 drug, 19 placebo)	Severity (on 0–5 scale) 0 = 6 participants 1 = 2 participants 2 = 6 participants 3 = 3 participants 4 = 1 participant 5 = 2 participants	Post-stroke (2–8 weeks)	Levodopa group showed significantly greater improvements in repetition and verbal fluency than placebo group. No significant differences between groups on other BDAE subtests. Anterior lesions (motor aphasia) associated with greater improvement.
**Acetylcholinesterase inhibitors**						
Berthier et al. (2006)	Donepezil, 10 mg daily for 16 weeks	Double- blind, randomized, placebo- controlled, parallel group, SLT was 2 h/week	*N* = 26 (13 drug, 13 placebo)	(mean WAB AQ for donepezil group 62.6 ± 23.8; range 13.6–90.4; mean placebo group 59.5 ±15.2; range 33.4–77.8)	Unilateral stroke lesion, chronic aphasia > 1 year	Donepezil group showed significantly greater improvement in WAB AQ and naming (PALPA) when compared with placebo group.
Berthier et al. (2014)	Donepezil, 10 mg daily	Open label, crossover with distributed therapy first and then massed therapy each for 40 hours	*N* = 3	Chronic conduction aphasia	Large left perisylvian infarcts	All three participants used donepezil and showed improved performance after both massed and distributed practice
Pashek and Bachman (2003)	Donepezil, 5 mg daily for 6 weeks	Open-label case study	*N* = 1	Broca's aphasia	7 months post stroke	Improvements in language, cognition, and motor speech following SLT + donepezil
Zhang et al. (2018)	Donepezil	Meta-analysis of five studies	*N* = 277	^*^Experimental: 102 Broca's, 13 Wernicke's, 1 Conduction, 13 Anomic, 9 Global; Control: 103 Broca's, 16 Wernicke's, 3 Conduction, 8 Anomic, 10 Global.	Post-stroke	Donepezil use improved auditory comprehension, naming, repetition and oral expression significantly greater than control groups.
Woodhead et al. (2017)	Donepezil, 5 mg up titrated to 10 mg	Double-blind, placebo-controlled, crossover, block randomization	*N* = 20	Group 1 (drug, then placebo): 7 moderate Wernicke's, 2 moderate Global, 2 severe Global; Group 2 (placebo, then drug): 3 moderate Wernicke's, 1 moderate Global, 1 severe Wernicke's, 4 severe Global	Post-stroke; chronic	Donepezil had a negative effect on comprehension in patients with moderate to severe Wernicke's or Global aphasia.
Hong et al. (2012)	Galantamine 8 mg up-titrated to 16 mg	Case-control, open label	*n =* 45 (23 drug, 22 placebo)	Galantamine group mean WAB AQ (Korean) 48.5 ± 27.4 Placebo group mean WAB AQ 54.3 ± 33.8	Post-stroke, chronic	Significant improvement on WAB AQ in galantamine group, but not in placebo group. Subcortical dominant lesion was predictive of responsiveness to galantamine.
**Glutaminergic medications**						
Barbancho et al. (2015)	Memantine, up-titrated to 10 mg twice daily	Randomized, double-blind, placebo-controlled, parallel- group trial EEG data from Berthier et al. (2009).	*n* = 28 (14 drug, 14 placebo) *n* = 15 healthy controls	Memantine group: 9 anomic, 4 Broca's, 1 Placebo group: 5 anomic, 4 Broca's, 2 conduction, 2 transcortical motor, 1 Wernicke's	Post-stroke, chronic	Memantine group showed bilateral neural reorganization on EEG, specifically related to N400 component. Healthy control participants showed no significant changes in their ERP patterns across the same period. Memantine + CILT had greater ERP changes corresponding to language changes than either treatment alone.
Berthier et al. (2009)	Memantine, up-titrated to 10 mg twice daily	Randomized double-blind, placebo-controlled, parallel-group	*n* =14 drug *n* = 14 placebo (13 completed)	Memantine group: 9 anomic, 4 Broca's, 1 Placebo group: 5 anomic, 4 Broca's, 2 conduction, 2 transcortical motor, 1 Wernicke's	Post-stroke, chronic	Memantine alone resulted in improved WAB, greater than placebo alone. When 2 weeks of CILT was added, the memantine group showed the greatest improvements, which was maintained at 2-week follow up.
Gawande et al. (2024)	Memantine, up-titrated to 15 mg for 6 weeks and then discontinued due to headache as a side-effect	Case study	*n* = 1	Global aphasia (improved to Broca's aphasia after treatment)	16 months post-stroke	WAB-AQ improved from 12 to 35 6 weeks after treatment. Improved comprehension
Zhang et al. (2018)	Memantine	Meta analysis of four studies	*N* = 124	^*^Experimental: 36 Broca's, 4 Wernicke's, 3 Conduction, 9 Anomic, 3 Global; Control: 33 Broca's, 6 Wernicke's, 3 Conduction, 5 Anomic, 3 Global.	Post-stroke	Significant differences in naming, spontaneous speech, and repetition for memantine group greater than control group.
**Nootrophic medications**						
Orgogozo (1999)	Piracetam		*N* = 12	Acute aphasia	Post-CVA	10% more people in the piracetam group recovered from aphasia within 12 weeks than in the placebo. When analyzing data from only people who received piracetam within the first 7 h post-stroke, 16% more people recovered in the piracetam group than placebo within 12 weeks.
Kessler et al. (2000)	Piracetam 2,400 mg twice a day	Intensive ST for 6 weeks	*n* = 12 drug *n* = 12 placebo	Mild-moderate aphasia	Up to 14 days post left ischemic stroke; lesion location in each group (5 frontal, 3 subcortical, 4 temporal)	Greater improvements in language measures and increased blood flow in language cortex via PET in individuals taking piracetam than placebo.
Zhang et al. (2016)	Piracetam	Meta-analysis of seven studies	*N* = 261	Mild-severe	4–24 weeks post-stroke (ischemic or hemorrhagic)	No significant effect of piracetam on aphasia severity.

* Original papers reported percentages across studies. Discrepancies of 1–2 participants are a result of converting rounded percentages to number of participants. CVA, cerebrovascular accident; WAB, Western Aphasia Battery; AQ, aphasia quotient; NR, not reported; MCA, middle cerebral artery; BDAE, Boston Diagnostic Aphasia Examination; SSRI, selective serotonin reuptake inhibitor; IFG, inferior frontal gyrus; SLT, speech and language therapy; CILT, constraint induced language therapy; ERP, event-related potential; PET, positron emission tomography.

*SNRIs.* There is emerging evidence in a small sample of four individuals with “motor-dominant” aphasia, that atomoxetine (i.e., Strattera) may assist with language recovery post-stroke (Yamada et al., 2016). Atomoxitine combined with intensive speech therapy resulted in improved language ability and increased blood flow on SPECT in the left inferior frontal gyrus. Neuroimaging confirmed that improvements in language were associated with increased cerebral blood flow, pointing to the utility of SNRIs in modulating norepinephrine and dopamine to increase neuroplasticity. Additional research is needed to replicate these preliminary findings.

##### Amphetamines

Amphetamines have been studied as an aphasia therapy adjuvant with inconsistent findings. Whereas, there is some evidence for benefits of amphetamines (Keser et al., 2017; Walker-Batson et al., 2001), there has been other research that has shown no significant improvement (McNeil et al., 1997).

It should be noted that most of the studies investigating dextroamphetamine as a treatment for post-stroke aphasia are small studies with modest results and lack of replication (Stockbridge, 2022). Although amphetamines have been studied for post-stroke aphasia, they are not commonly prescribed to individuals post-stroke due to the risk of increased blood pressure; however, several studies have found that when administered and monitored appropriately, there may not be a significant risk of hypertension with the use of dextroamphetamine post-stroke (Keser et al., 2017; Walker-Batson et al., 2001)

##### Dopamine agonists

Dopamine agonists have been studied for aphasia treatment with inconsistent findings. Bromocriptine in high dosages was shown to improve reading comprehension and repetition in 11 individuals with chronic non-fluent aphasia (Bragoni et al., 2000) however, there are multiple studies that found no significant gains with its use in the treatment of aphasia (Ashtary et al., 2006; Gupta et al., 1995; Sabe et al., 1995). Similarly, levodopa has very modest support from one randomized controlled trial (Seniów et al., 2009) which showed significantly greater improvements in repetition and verbal fluency abilities over placebo. Individuals with anterior lesions tended to show greater improvement, which suggests that lesion location may play a role in its efficacy for post-stroke aphasia.

One caveat to the use of dopamine agonists is that there has been some discussion of the role of genetics and the ability to benefit from cortical plasticity vs. subcortical plasticity, which could, in theory, be enhanced by dopamine agonists. Individuals with the atypical variant of BDNF (i.e., -met allele carriers) show less ability to benefit from cortical plasticity induced by tDCS and aphasia therapy (Fridriksson et al., 2018b; Kristinsson et al., 2019). Di Piño et al. (2016) suggest that subcortical plasticity should be investigated in -met carriers, potentially via dopamine agonists, to determine if they are more likely to respond, based on studies of mice (Qin et al., 2011, 2014). That is, individuals with the atypical variant of BDNF may rely more on subcortical plasticity since cortical plasticity is disrupted. This highlights the likely possibility that genetics may modulate best candidacy for medications used to enhance neuroplasticity.

#### Cholinergic medications

##### Acetylcholinesterase inhibitors

In the chronic phase of recovery, donepezil is the most studied AChEi in individuals with post-stroke aphasia and has a good amount of support for its efficacy. Several clinical trials have shown that donepezil may enhance language recovery in chronic post-stroke aphasia, particularly when combined with speech and language therapy (Berthier et al., 2006, 2014; Pashek and Bachman, 2003; Yoon S. Y. et al., 2015). In a double-blind, randomized, placebo-controlled trial, Berthier et al. (2006) found a large effect size for improvement in overall language function (WAB AQ) and naming abilities (PALPA) in 13 participants who used donepezil when compared with 13 individuals who received placebo. Aphasia therapy occurred for 2 h per week and was a “syndrome-specific standard approach” (Berthier et al., 2006; p. 1687). Zhang et al. (2018) conducted a meta-analysis of five studies that included a total of 277 patients. Donepezil was found to be beneficial for post-stroke aphasia. However, one study found that donepezil may have worsened comprehension in patients with moderate to severe chronic Wernicke's or global aphasia (Woodhead et al., 2017), indicating there may be an interaction with aphasia severity or type. In sum, donepezil has a good amount of evidence supporting its use as an adjuvant to post-stroke language therapy.

Evidence for galantamine in post-stroke aphasia is less extensive. A review of 96 patients treated with galantamine in the chronic period and 60 in the acute phases showed that use of galantamine or donepezil yielded positive effects in spontaneous speech, repetition, naming, and auditory comprehension (Dávila et al., 2023). Moreover, one case-control study found that galantamine significantly improved language function in patients with chronic post-stroke aphasia as measured by the Western Aphasia Battery (Hong et al., 2012). Thus, there is some evidence for efficacy of AChEi medications in the treatment of aphasia.

#### Glutaminergic medications

Various studies (Barbancho et al., 2015; Berthier et al., 2009; Cichon et al., 2021; Gawande et al., 2024) suggest that memantine may be a safe and effective adjuvant to aphasia therapy in the chronic stage of recovery. One clinical trial (Berthier et al., 2009) found that treatment with memantine alone resulted in improved aphasia severity scores over placebo. The addition of 2 weeks of constraint-induced language therapy yielded highly significant improvements from both groups, with the memantine + therapy group showing the greatest improvements. Barbancho et al. (2015) analyzed electroencephalography (EEG) data from the same trial and found that individuals in the memantine group demonstrated bilateral neural reorganization, specifically related to the N400 component, which is associated with semantic processing. They also demonstrated that the combination of memantine and constraint-induced language therapy yielded greater ERP changes over memantine or placebo alone.

#### Nootropic medications

The research on piracetam has yielded mixed results. Some studies show benefits (Stockbridge, 2022), whereas others report no significant advantages (Huber et al., 1997; Zhang et al., 2018). The Piracetam in Acute Stroke Study (PASS) found that for individuals with aphasia, 10 percent more patients in the piracetam group recovered from aphasia within 12 weeks than in the placebo group. When analyzing data from only those individuals who received piracetam in the first 7 h, the difference between groups increased to 16% (Orgogozo, 1999). Corroborating this work, Kessler et al. (2000) found greater improvements in language measures and increased blood flow in language eloquent cortex in individuals taking piracetam over placebo. However, a later meta-analysis on piracetam for post-stroke aphasia found no significant effect of piracetam on aphasia severity (Zhang et al., 2016). Thus, early pilot studies endorsed some advantages to the use of piracetam in this population, but follow-up meta-analyses failed to demonstrate efficacy.

In sum, donepezil and memantine are two medications with the strongest support in the literature for the treatment of aphasia (Cichon et al., 2021). Galantamine also has emerging evidence (Dávila et al., 2023; Hong et al., 2012). The degree to which SSRIs improve language is unclear, but they are commonly used for depression, which is prevalent post-stroke, and thus, could also have an indirect effect on language. There has been little support for dopamine agonists, such as bromocriptine and levodopa in the treatment of post-stroke aphasia; however, future research taking genetics into consideration, specifically BDNF polymorphisms may assist with matching patients who may be more or less responsive. Any potential gains from pharmacological adjuvants should be weighed against medication side effects.

Future research should investigate the best choice of medications based on individual profiles, such as aphasia presentation, cognitive skills, co-morbidities, and biomarkers. Future research should also explore the combination of various monoaminergic medications or the combination of monoaminergic and cholinergic medications as an adjuvant to aphasia therapy. Larger scale randomized controlled trials should be conducted to establish treatment guidelines, including dosing and treatment duration. Finally, the promise of these medications in combination with non-invasive brain stimulation techniques should continue to be investigated.

There are other techniques that have been explored for aphasia treatment that are compensatory in nature (e.g., brain-computer interface, AAC) or may be used for restorative therapy (e.g., virtual reality), but they are not adjuvants as we have defined herein. That is, they may be a vehicle for enhanced communication or a therapeutic technique, but they are not used to create a more neuroplastic brain environment as a canvas for the experience of therapy. We also opted to exclude yoga, even though it is gaining popularity as a therapeutic technique for aphasia, as the mechanism remains unclear. See the following references for more information on brain computer interface (Kleih and Botrel, 2024; Musso et al., 2022), AAC (Dietz et al., 2020), virtual reality (Devane et al., 2023), and yoga (Bislick et al., 2023).

## Discussion

Adjuvant techniques offer promising ways to enhance aphasia therapy outcomes by several different mechanisms of action. Non-invasive brain stimulation (NIBS), aerobic exercise, and pharmacological interventions may contribute to a more favorable brain environment that supports neuroplasticity, which is essential for effective language recovery. NIBS techniques, such as TMS and tDCS, can modulate brain activity to improve language processing. For example, improved naming abilities have been observed in studies where rTMS (Yao et al., 2020; Yoon T. H. et al., 2015) and tDCS (Fridriksson et al., 2018b; Zheng et al., 2024) were combined with SLT. Future studies are needed to clarify the duration of effects, the optimal stroke stage for stimulation, and which stimulation technique or parameters are favored depending on patient-specific factors.

Aerobic exercise can increase levels of BDNF, a neurotrophin that promotes neurogenesis and is important for cognitive and language function. BDNF plays a critical role in neuroplasticity and is known to be important for learning and memory. Depression (Pompon et al., 2019, 2022), stress (Jewell and Harnish, 2024), and anxiety are common challenges in post-stroke aphasia rehabilitation. Exercise has the potential to mitigate these effects as well (Harris et al., 2019).

Pharmacotherapies that target neurotransmitter systems can improve cognitive functions and mood, further supporting therapy engagement. For example, memantine, an NMDA receptor antagonist that is considered a glutaminergic medication, has resulted in significant improvements in language function in individuals with chronic post-stroke aphasia, particularly when combined with constraint-induced language therapy (Barbancho et al., 2015; Berthier et al., 2009). Donepezil, an acetylcholinesterase inhibitor, has also shown promise in some studies when combined with SLT, although there is some evidence of negative effects on comprehension for moderate to severe aphasia (Woodhead et al., 2017). Individualizing pharmacotherapies based on an individual's specific needs is crucial. Future studies are needed to determine the optimal drug and language treatment regimen based on an individual's probability of responsiveness.

The effectiveness of adjuvant techniques likely vary depending on individual factors, such as the type and severity of aphasia (Woodhead et al., 2017), the time since stroke, lesion size and location (Seniów et al., 2009), genetics (Di Piño et al., 2016; Qin et al., 2011, 2014), and overall health status. A comprehensive approach that combines evidence-based aphasia therapy with individualized adjuvant techniques has the best chances of improving outcomes for individuals with aphasia.

## Footnotes

^1^Evidence of efficacy sections include a table summarizing the experimental trials reviewed. These tables include the aphasia subtypes reported in each trial to provide a clearer understanding of which individuals may benefit from the adjuvant technique best. Importantly, this paper did not conduct a systematic review. The articles included in this review and corresponding tables are not an exhaustive list of all completed trials for the adjuvant technique.
